# Electrochemical mineralization of iron-tannate stain on HAp and bovine enamel-A non-peroxide approach

**DOI:** 10.1016/j.heliyon.2021.e07296

**Published:** 2021-06-12

**Authors:** Vembu Suryanarayanan, Deepak Kumar Pattanayak, Rethinam Senthil Kumar, LaTonya Kilpatrick, Suman Chopra, GuoFeng Xu, Lin Fei, Cajetan Dogo-Isonagie, Patrik Johansson

**Affiliations:** a Electro Organic & Materials Electrochemistry Division, CSIR-Central Electrochemical Research Institute, Karaikudi, 630003, TamilNadu, India; b Electrochemical Process Engineering Division, CSIR-Central Electrochemical Research Institute, Karaikudi, 630003, TamilNadu, India; c Atotech Development Center Private Limited, Gurgaon, Haryana, India; d Colgate-Palmolive Company, Oral Care Product Development, 909 River Road, Piscataway, NJ, 08854, USA

**Keywords:** Hydroxyapatite (HAp), Bovine enamel (BE), Hypochlorite, Iodine, Hydrogen peroxide

## Abstract

Prolonged treatments for the destaining of teeth using high concentrations of hydrogen peroxide may cause secondary unwanted effects such as tooth hypersensitivity and gingival irritation. Hence, it is aimed to develop a non-peroxide-based method to oxidize iron-tannate (Fe-TA) stained hydroxyapatite (HAp) and bovine enamel (BE) samples. Constant current electrolysis (CCE) experiments were carried out on Pt working electrode in aqueous NaCl, KCl and KI solutions at discrete concentrations under continuous experiment and a non-continuous experiment. CCE shows that in the presence of iron tannate (Fe-TA) stained HAP, approximately 30 ppm of iodine was generated using 0.1M KI and nearly 40 ppm was produced with 0.2 M KI. By using a non-continuous CCE process, the lowest amount of chlorine was generated from NaCl solution, which was well within the safety limits for oral applications. Depending on the experimental conditions used, between 13 ppm and 124 ppm of chlorine was generated. CCE of Fe-TA stained on HAp using KCl reveals that at the lowest current density of 10 mA/cm^2^, the amount of hypochlorite generated was 20 ppm on Pt electrode having a surface area of 6 cm^2^. Ion chromatographic (IC) analysis revealed that non-continuous CCE of Fe-TA-BE in NaCl generated a low concentration of sodium perchlorate (0.8 ppm), whereas the continuous process generated no perchlorate, but a considerable higher quantity of chlorate for Fe-TA-BE (37 ppm) and Fe-TA-HAp (140 ppm) samples.

## 1 Introduction

Tooth color is a cosmetic benefit and white teeth are regarded as an important component of personal hygiene and overall beauty. However, lifestyle choices such as the frequent consumption of tea, wine, and coffee or chewing or smoking tobacco can lead to tooth discoloration. Tannic acid (TA) is a water-soluble polyphenol, generated by a glucose moiety substituted by gallic acid dimers; and it produces a brown colored solution when dissolved in water containing a source of iron ions. Polyphenols are vulnerable to oxidation, as they need oxidation for most of their biological activity (e.g., antimicrobial) [1]. Further, they behave as antioxidants owing to their radical scavenging and metal chelating activity [2] . Ordinary black tea is rich in TA and is even more aggressive at staining teeth than coffee, which has lots of chromogens. TA promotes staining by enhancing the ability of chromogens to stick to enamel, leading to high-coloration of the tooth. Subsurface enamel stains occur when the staining compounds diffuse and accumulate within the deeper enamel region.

Iron is most normally found in nature in the form of an oxide. Iron, as Fe^2+^ ions with a concentration of 40 μg/L, can be identified by taste in refined water. Mineralized spring water can contain up to 500 mg/L of Fe^2+^ ions. The estimated taste limit is 0.12 mg/L. In well-water, the concentration of iron is less than 0.3 mg/L and is not generally detectable by taste, whereas the acceptable taste levels are 0.3–3 mg/L [3]. The combined intake of tannins from different food samples and iron from drinking water contributes to the formation of iron tannate which could be a component of tooth stains [4].

Teeth whitening has become an important cosmetic treatment, leading to the generation of millions of dollars per year [5]. The traditional method to whiten teeth is by bleaching with hydrogen peroxide (HP) or carbamide peroxide (CP). CP is composed of hydrogen peroxide and urea at different concentrations (0.1–35%) [6]. Hydrogen peroxide is particularly effective at whitening teeth as it not only bleaches chromophores on the surface of teeth by oxidizing the chromophore molecule to a colorless one, but hydrogen peroxide is also able to diffuse into enamel and bleach subsurface (intrinsic) stains as well. The rate and effectiveness of teeth whitening treatments improve with increased concentrations of hydrogen peroxide [7]. However, the regulatory restrictions in many regions limit the level of hydrogen peroxide allowed in over the counter dental products and prolonged treatments using high concentrations of hydrogen peroxide may cause secondary unwanted effects such as tooth hypersensitivity and gingival irritation [8]. As a result, the development of effective, non-peroxide tooth whitening technologies is an active area of research in the oral care industry. In this report, an electrochemical technique is described as an eco-friendly method for the effective removal of stains from teeth.

In the electrochemical process, the organics are destroyed by either the direct or indirect oxidation process, where free radicals formed break the aromaticity of the staining molecules, resulting in colorless products [9, 10]. In a direct anodic oxidation process, the materials adsorbed on the anode surface are destroyed by the anodic electron transfer reaction. Since the tooth models involving hydroxyapatite (HAp) and bovine enamel (BE) are electrochemically inactive, the direct method is not considered to be suitable for the present work. On the other hand, in an indirect oxidation process, electrogenerated oxidizing species (EOS) such as hypochlorite/chlorine are electrochemically generated, which can oxidize organic molecules in the bulk solution [11, 12, 13]. Among the different oxidants, hypochlorite has been considered for indirect oxidation since the precursor chloride salts are not toxic, inexpensive and easily available [14, 15]. The active role of hypochlorite has been extensively investigated in the literature for dye degradation [16, 17, 18, 19, 20]. Various literature reviews concerning the electrochemical oxidation of various organic materials and pollutants in waste water treatments had been well documented [21, 22, 23, 24, 25, 26, 27, 28, 29, 30, 31, 32, 33, 34].

The above literature surveys clearly suggest that the principle on the generation of an oxidizing species by electrochemical process and their application on the destruction of organic molecules can also be applied for the removal of stain formed on the tooth models, which may be an alternative to the present method of peroxide treatment. Till now, there is only one method for the teeth whitening involving on the application of sodium metabisulphite as a reducing agent [5] and no other strategies involving either chemical or electrochemical are adopted for the stain removal studies. The present investigation is proposed to analyze about (i) Effective oxidation of iron tannate in solid phase by the generated iodide or hypochlorite ion in solution phase, compared to hydrogen peroxide, (ii) Influence of concentration of chloride or iodide salt, geometry of the working electrode and mode of operation of galvanostatic electrolysis with suitable current density on the sufficient generation of hypochlorite ionic species towards stain removal on HAp and BE disks.

Hence, the objective of the present investigation is aimed to develop an electrochemical procedure, for the first time, on the destaining of the HAp and BE tooth models decorated with Fe-tannate as a model stain. The *in vitro* electrochemical whitening was carried out on platinum electrode in different aqueous supporting electrolyte salts, namely KI, NaCl and KCl at various current densities both in discontinuous as well as in continuous mode of constant current electrolytic procedure. It is well known that the tooth is made up of hydroxyapatite with the formula Ca_10_(PO_4_)_6_(OH)_2_, where in acidic pH condition, the hydroxyapatite dissolves, leading to the formation of Ca salts. Further, previous literature clearly confirms that chlorine evolution predominates at acidic pH values, while HOCl leads in the range from 2.5 < pH < 7.5, followed by the dominant OCl− at alkaline conditions [34]. In this study, HOCl is chosen as oxidant for the destaining process, which is being generated at neutral pH, and as a result, the electrolysis was conducted only at pH of 7. Further, all the investigations were carried out at room temperature since the practical destaining procedure in the clinical trials adopts optimum human body temperature. The amount of loading of iron tannate on HAp and BE tooth models were provided by Colgate Palmolive, USA, which was optimized for their oral formulation. A suitable mechanism involving the formation of different oxidizing species during the course of electrolysis is also discussed.

## 2 Materials and methods

### 2.1 Synthesis of HAp powder and staining of different tooth models

The HAp ceramic powder was prepared by a solution route as per an earlier described procedure [35, 36, 37]. The samples were pre-treated in a pH adjusted solution of artificial saliva (the composition of artificial saliva is given in Table 1 where solution A and B is mixed in 1:1 ratio) for 3 h (pH 7.4) and then immersed in 0.001M tannic acid for 30 min, followed by immersion in 0.001 M ferrous sulphate solution for 10 min and finally equilibrated in artificial saliva for 10 min. This procedure was repeated four times per day for five days (rinsing the substrate with water only after its removal from artificial saliva).

**Table 1:** Composition of artificial saliva.

No.	Name of the compound	wt (%)
**Solution A**		
1	Deionised water	98.95
2	Ammonium chloride	0.046
3	Potassium chloride	0.231
4	Potassium hydrogen phosphate	0.071
5	Sodium bicarbonate	0.4
6	Sodium phosphate	0.08
7	Urea	0.035
8	Calcium chloride	0.04
9	Magnesium chloride hexahydrate	0.009
10	Potassium thiocyanate	0.044
11	Sodium citrate dihydrate	0.002
12	Bovine serum albumin (BSA)	0.005
13	Glycine	0.090
**Solution B**		
1	Deionised water	97.5
2	Mucin type II	2.5

### 2.2 Electrochemical experiments

**Figure 1:**
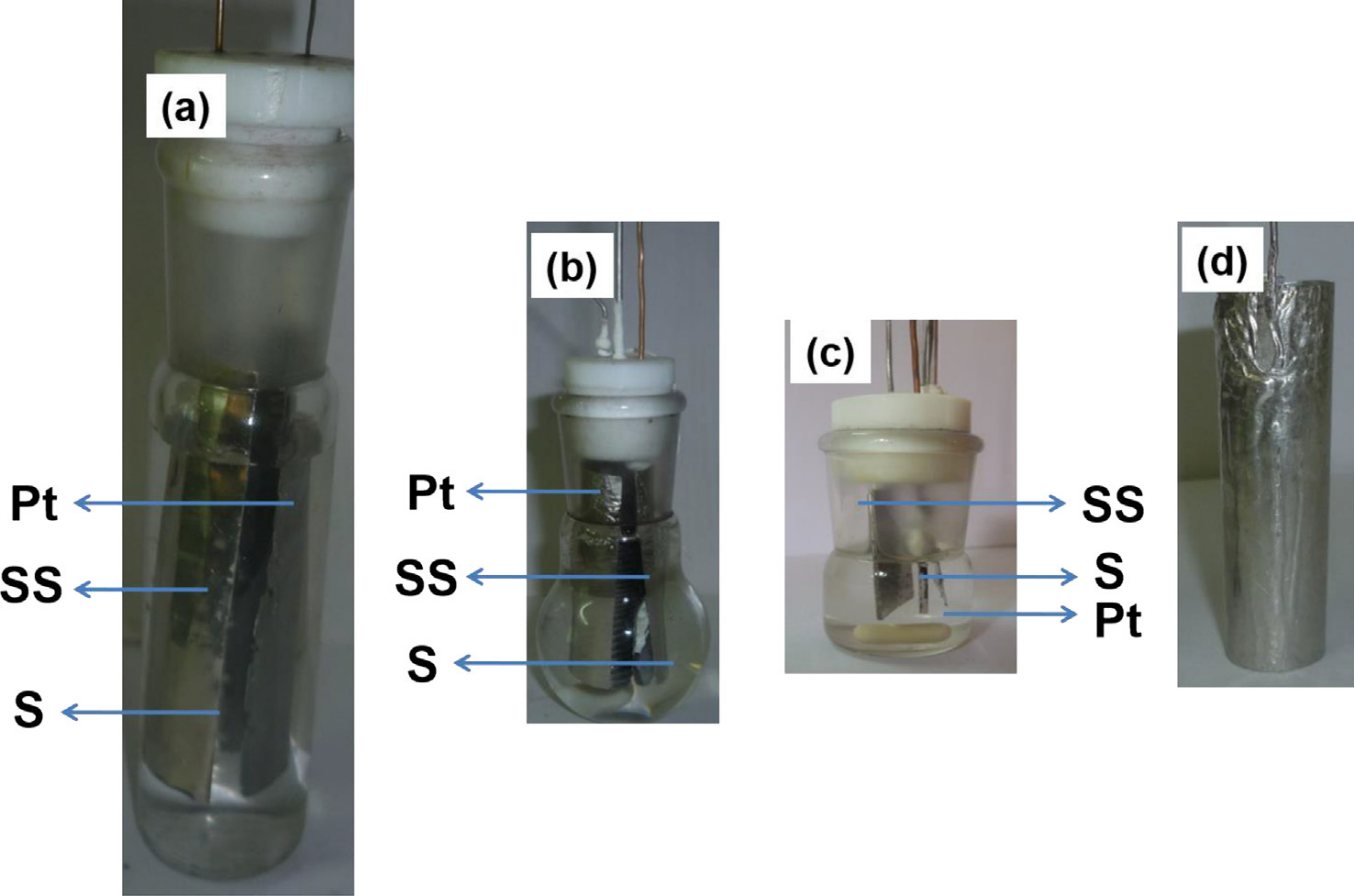
Electrochemical cell with Pt area of a) 16 cm^2^, b) 6 cm^2^, c) 1 cm^2^, d) Cylindrical Pt electrode with the area of 16 cm^2^: SS represents stainless steel counter electrodes and S belongs to sample.

An undivided electrochemical cell was used with a volume capacity varying from 10 to 15 cm^3^ of electrolyte solution. Constant potential electrolysis (CPE) experiments were carried out with the cell containing three electrodes namely Pt working (1 cm^2^), stainless steel counter and Pt reference. Platinum is a relatively inert metal which does not corrode easily and it has catalytic qualities on various redox electrochemistry. Constant current electrolysis (CCE) was conducted using either Pt foils having areas of 1 and 6 cm^2^ or a Pt cylinder with an area of 16 cm^2^. For the CCE experiment using KI, 1 cm^2^ area of Pt was used. An example of the electrochemical cell with different types of Pt electrodes is shown in Figure 1. Some CCE experiments were carried out by changing the electrolyte solution every ten minutes (non-continuous process) and the hypochlorite generated was estimated subsequently (See Section 2.3). Other CCE experiments were conducted without changing the electrolyte solution (continuous process). For both types of experiments, different concentrations of NaCl (0.06 M and 0.1 M), KCl (0.1 M–0.4 M), and KI (0.1 M–0.2 M) were used. For the CPE experiments, the pH was adjusted between acid (pH = 5), neutral (pH = 7), and alkali (pH = 9). For the CCE experiments, the pH was kept at neutral condition.

### 2.3 Estimation of chlorine and iodine in the form of hypochlorite

Chlorine, the reaction product, was estimated by oxidizing the iodide ion (from KI solution) to free iodine using hypochlorite (ClO^−^) and then titrating the formed iodine with standardized sodium thiosulfate solution. Similarly, iodine was estimated, by titrating against a standardized sodium thiosulfate solution. The exact procedure for the titration and calculations for finding the amount of chlorine and iodine is given as follows:

Chlorine water cannot be titrated against the standard thiosulfate solution because of the difficulty on the detection of the end point. The standard procedure is to react the chlorine water with an excess of acidified KI which converts I^−^ ion to I_2_ by ClO^−^ ion which can be detected easily [38, 39] (Equation 1).

(1) ClO^−^ + 2I^−^ + 2 H^+^ → I_2_ + Cl^−^ + H_2_O

The formed I_2_ product is titrated with thiosulfate. When acidified KI is added, the solution turns yellow. To this solution, starch is added, where a blue color is formed. This, on titration with thiosulfate solution, leads to the disappearance of blue color revealing the end point [38, 39] (Equation 2).

(2) 2 S_2_O_3_^2-^ + I_2_ → 2 I^−^ + S_4_O_6_^2-^

**Step 1 - Standardisation of sodium thiosulfate against potassium dichromate**

Sodium thiosulfate solution was standardised with acidified K_2_Cr_2_O_7_ solution containing 10% KI. The evolved iodine during the course of reaction was estimated using starch as indicator [38, 39] (Equation 3).

(3) K_2_Cr_2_O_7_+ 6KI + 7H_2_SO_4_ → Cr_2_(SO_4_)_3_+ 4K_2_SO_4_ + 3I_2_+ 7H_2_O

The method involves the preparation of 0.02N potassium dichromate solution followed by standardization of sodium thiosulfate solution.

At first, burette was loaded with sodium thiosulphate solution and 10 ml aliquot of prepared standard potassium dichromate solution was pipetted out into a flask. To this, 10 ml of 10 % KI solution and 10 ml of 1 M H_2_SO_4_ solution was added. The flask was covered with a stopper and put into dark place at 10 min. The sample solution was titrated with thiosulfate until the solution becomes pale yellow. Then 5 drops of starch indicator were added to this and titrated with constant stirring till the blue color disappears (end point). The normality of the sodium thiosulphate solution was calculated accordance to equivalents law [40] (Equation 4),

(4) V_1_N_1_ = V_2_N_2_

where

V_1_ = Volume of potassium dichromate solution

N_1_ = Normality of potassium dichromate solution

V_2_ = Volume of sodium thiosulphate solution

N_2_ = Normality of sodium thiosulphate solution.

**Step 2 - Determination of chlorine concentration**

Aliquot of chlorine solution (2 ml) obtained from CCE was added to the flask. To the above solution 4 ml of acidified KI solution was added (yellow color). 2 drops of starch indicator were added and titrated with constant stirring till the blue color disappeared. The titration was repeated two times for getting concordance value. Strength of chlorine solution was calculated using sodium thiosulphate standard solution. The chlorine concentration in the CCE sample (in ppm) was estimated as follows [40] (Equation 5):

(5) $$\left(\frac{\left(\text{Volume of thio solution}\times \text{Strength of thio solution}\right)}{\text{Volume of chlorine solution}}\right)\times \text{Equivalent}\;\text{molar}\times 1000$$

Iodine was estimated at the end of CCE of KI, where it was titrated again standardized sodium thiosulfate and the other calculations following the estimation remain the same. The presence of chlorite, chlorate and perchlorate in samples at the end of CCE were confirmed by ion-chromatographic (IC) technique, carried out by Metrohm India Limited, Chennai.

### 2.4 Characterization

UV/Visible absorbance spectra were recorded on a VARIAN Cary 500 Scan spectrometer. Constant current electrolysis was carried out in an Aplab regulated DC power supply (Model L6410). Constant potential electrolysis was conducted on an electrochemical workstation (Eco Chemie Autolab Potentiostat system). Presence of chlorates and perchlorates were determined using Ion-Chromatography (930 Metrohm Combustion IC PP) with Metrosep A Supp 7–250/4.0 column by gradient elution with Eluent A (3.6 mmol/L sodium carbonate and 15% (v/v) acetone) and Eluent B (20 mmol/L sodium carbonate and 10% (v/v) acetone.

## 3 Results

### 3.1 Constant current electrolysis (CCE) in aqueous KI

**Figure 2:**
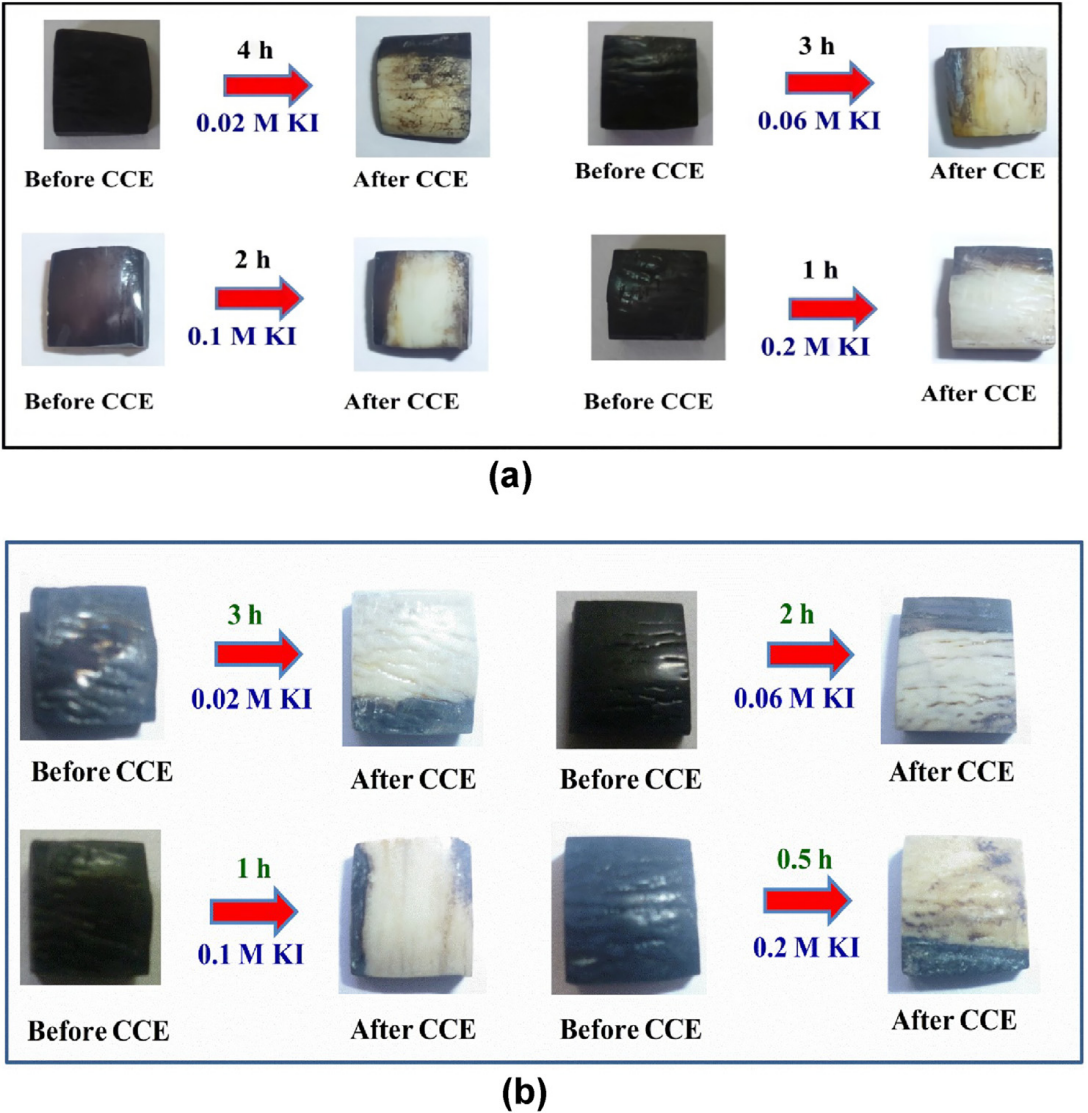
Photographic images obtained before and after CCE of Fe-TA-BEin phosphate buffer containing different concentrations of KI at current density of (a) 40 mA/cm^2^ & (b) 80 mA/cm^2^; surface area of Pt is 1.0 cm^2^.

**Figure 3:**
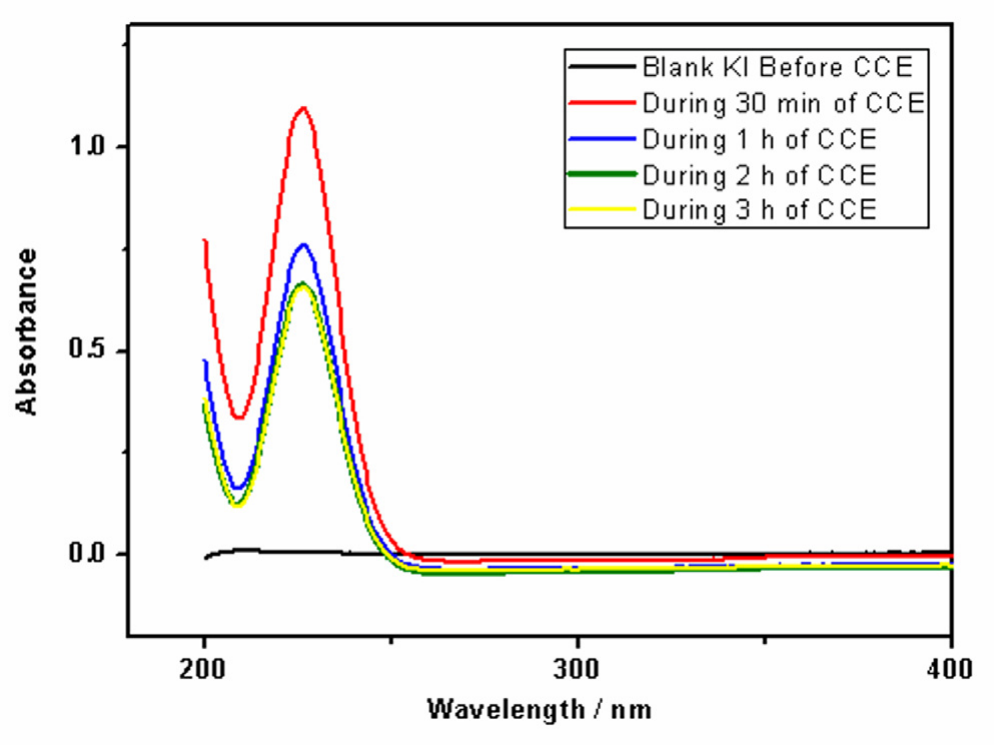
UV-Visible spectra of electrolyte solutions were taken at different intervals of time before and after CCE in phosphate buffer containing 0.1 M of KI.

CCE of iron tannate stained bovine enamel (Fe-TA-BE) tooth samples was carried out in phosphate buffer solution containing different concentrations (0.02 M, 0.06 M, 0.1M and 0.2 M) of KI at pH 7.4 on a Pt electrode (1 cm^2^) and the current densities were fixed at 40 (Figure 2a) & 80 (Figure 2b) mA/cm^2^. CCE experiments clearly indicate that less time is required for stain removal with increased electrolyte concentration (Figure 2). The pH of the electrolyte solution also increased from neutral to alkaline during the course of the reaction (see reaction mechanism in Figure 9). Using iodometric titration, 20 ppm of I_2_ was generated in 0.02 M KI, 25 ppm in 0.06 M KI solution, 30 ppm in 0.1M KI, and 40 ppm in 0.2 M KI. However, the efficiency of iodine generation to remove tooth stains was not very efficient since it took a long time for the complete removal of stains (2–3 h) and a yellow stain remained after the reaction on the BE sample. The increase in the resulting yellow stain was tracked using UV-Visible spectroscopy (Figure 3).

### 3.2 CCE in aqueous KCl and NaCl

**Figure 4:**
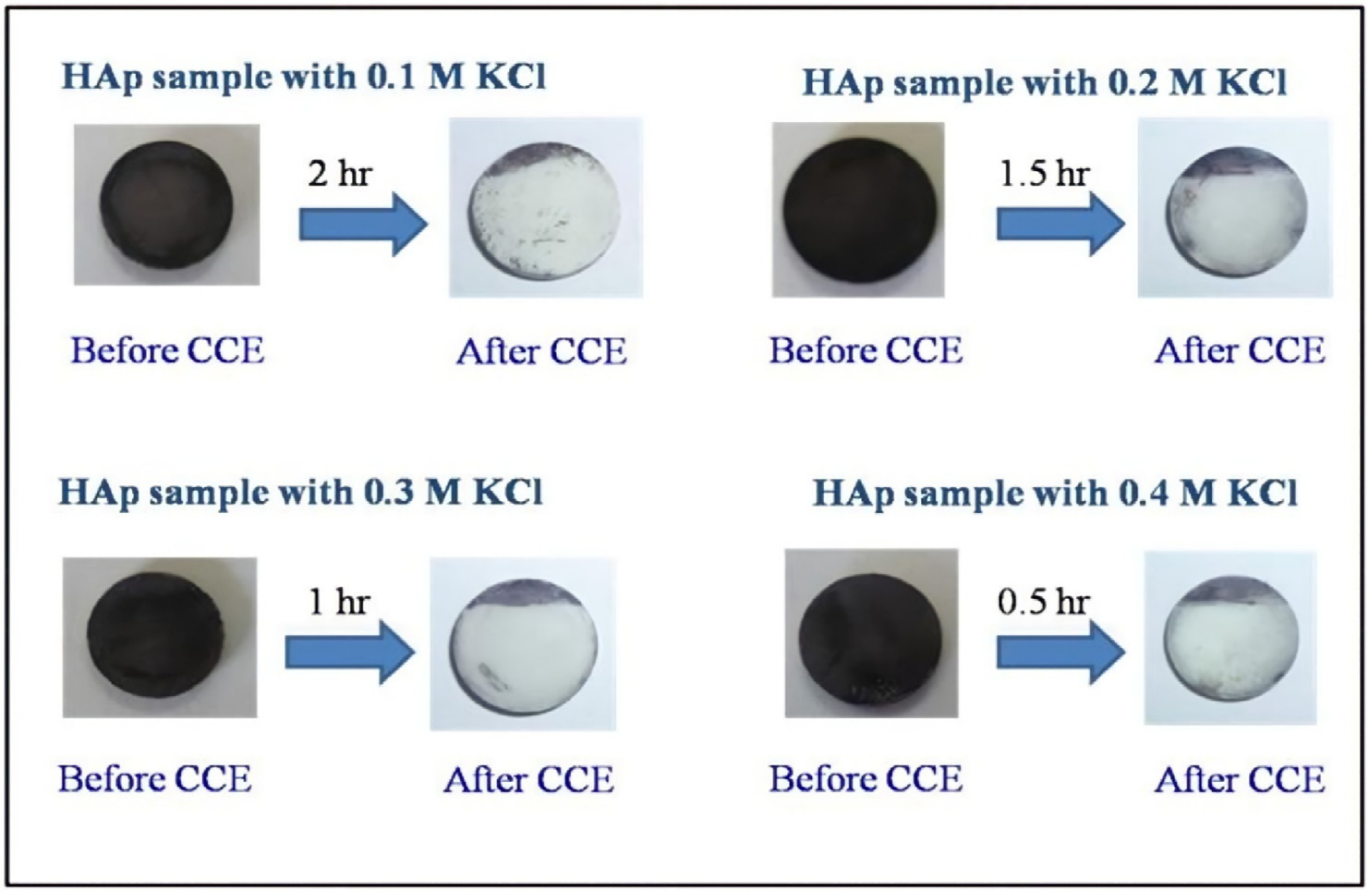
Photographic images obtained before and after CCE of Fe-TA HAp in phosphate buffer containing different concentrations of KCl. Surface area of Pt is 1.0 cm^2^.

**Figure 5:**
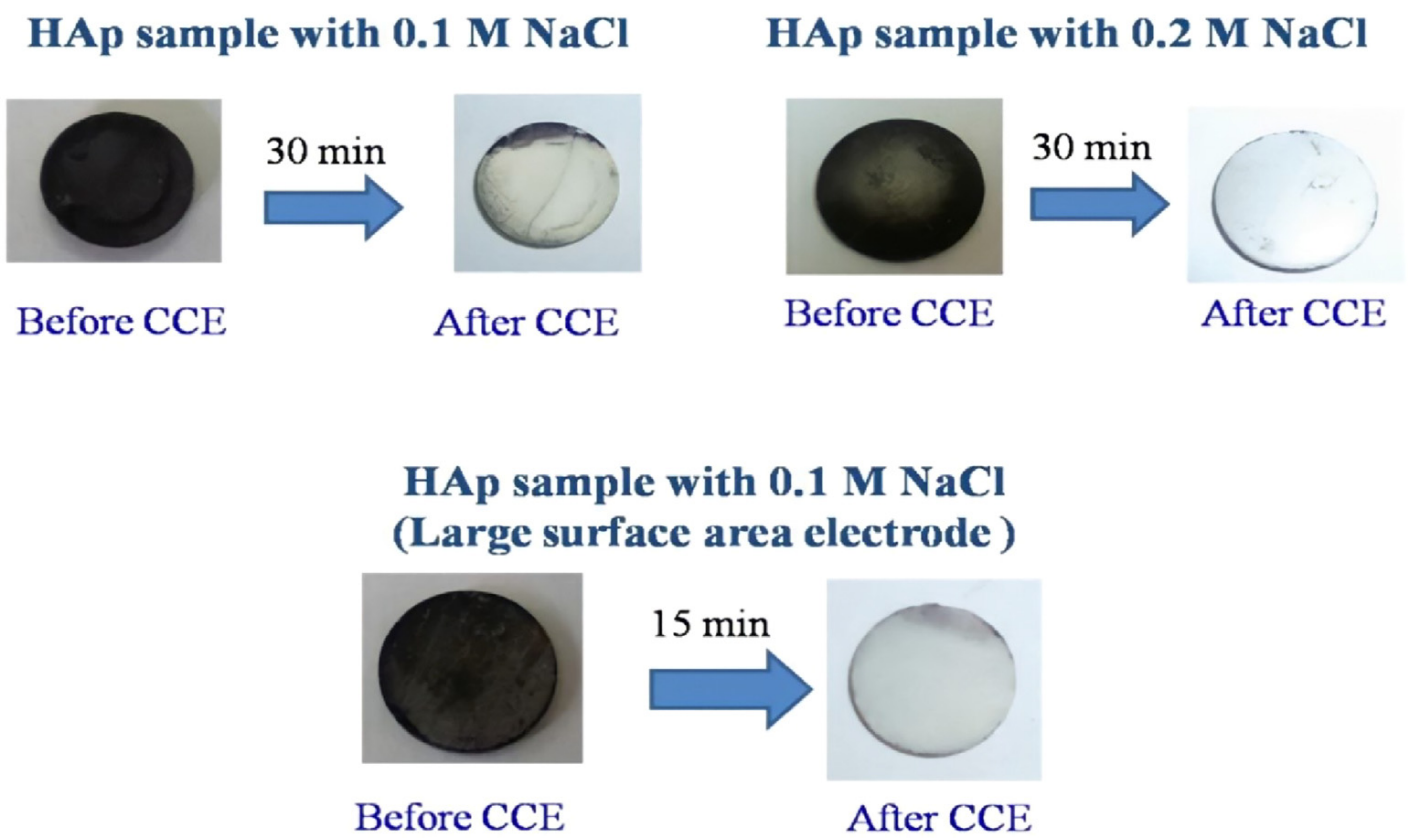
Photographic images obtained before and after CCE of Fe-TA HAp in phosphate buffer containing different concentrations of NaCl. Surface area of Pt is 1.0 cm^2^ for the first two images and 6 cm^2^ for the last image.

CCE of Fe tannate stained HAp was carried out using different concentrations of KCl and NaCl (0.1 M, 0.2 M, 0.3 M and 0.4 M) in phosphate buffer solution (pH 7.4). Figure 4 shows the photographic images obtained for CCE carried out on a Pt electrode (1 cm^2^). CCE carried out in 0.1 M KCl took 2 h to remove the stain. With an increase in the concentration of KCl from 0.1 to 0.4 M, the amount of time required to remove the stain decreased from 2 h to 0.5 h. The concentration of chlorine estimated for each CCE experiment is shown in Table 2. The estimated chlorine concentration for the CCE of Fe–Ta stained HAp was 35.5 ppm (0.1 M KCl), 70.9 ppm (0.2 M KCl), 88.6 ppm (0.3 M KCl) and 124 ppm (0.4 M KCl). The concentration of chlorine increased with increased KCl concentration and hence the time required to remove the stain was reduced. Unlike what was observed with KI, no color formed in solution and on the substrate during the electrolysis experiment. CCE studies were also carried out in different concentrations of sodium chloride solution to determine if there was a change in the efficiency of the stain removal process. Figure 5 shows the photographic images of CCE obtained in 0.1 and 0.2 M NaCl solutions. For both NaCl concentrations, only 30 min was required to remove the stain from Fe-TA-stained HAp using an applied current density of 80 mA/cm^2^. Consistent with the KI and KCl results, higher NaCl concentrations removed more of the stain. Table 2 lists the estimated amount of chlorine generated as a function of time for each NaCl concentration tested. The estimated chlorine content for 0.1 and 0.2 M NaCl after the CCE experiment was found to be (in ppm) 45 and 123 respectively (Table 2). This result reveals that the extent of chlorine evolution is more efficient in CCE experiments carried out in NaCl, when compared to KCl, resulting in more effective bleaching in shorter time duration.

**Table 2:** Amount of chlorine generated after CCE of Fe-TA-HAp with time duration for different concentrations of KCl and NaCl.

Sl No.	Nature of halide	Conc. of chloride (M)	Conc. Cl_2_ (ppm)	Time duration (hours)
1	KCl	0.1	35.5	2.0
2	KCl	0.2	70.9	1.5
3	KCl	0.3	88.6	1.0
4	KCl	0.4	124.0	0.5
5	NaCl	0.1	45.0	0.5
6	NaCl	0.2	123.0	0.5
7	NaCl (6 cm^2^)	0.1	88.6	15 min

**Figure 6:**
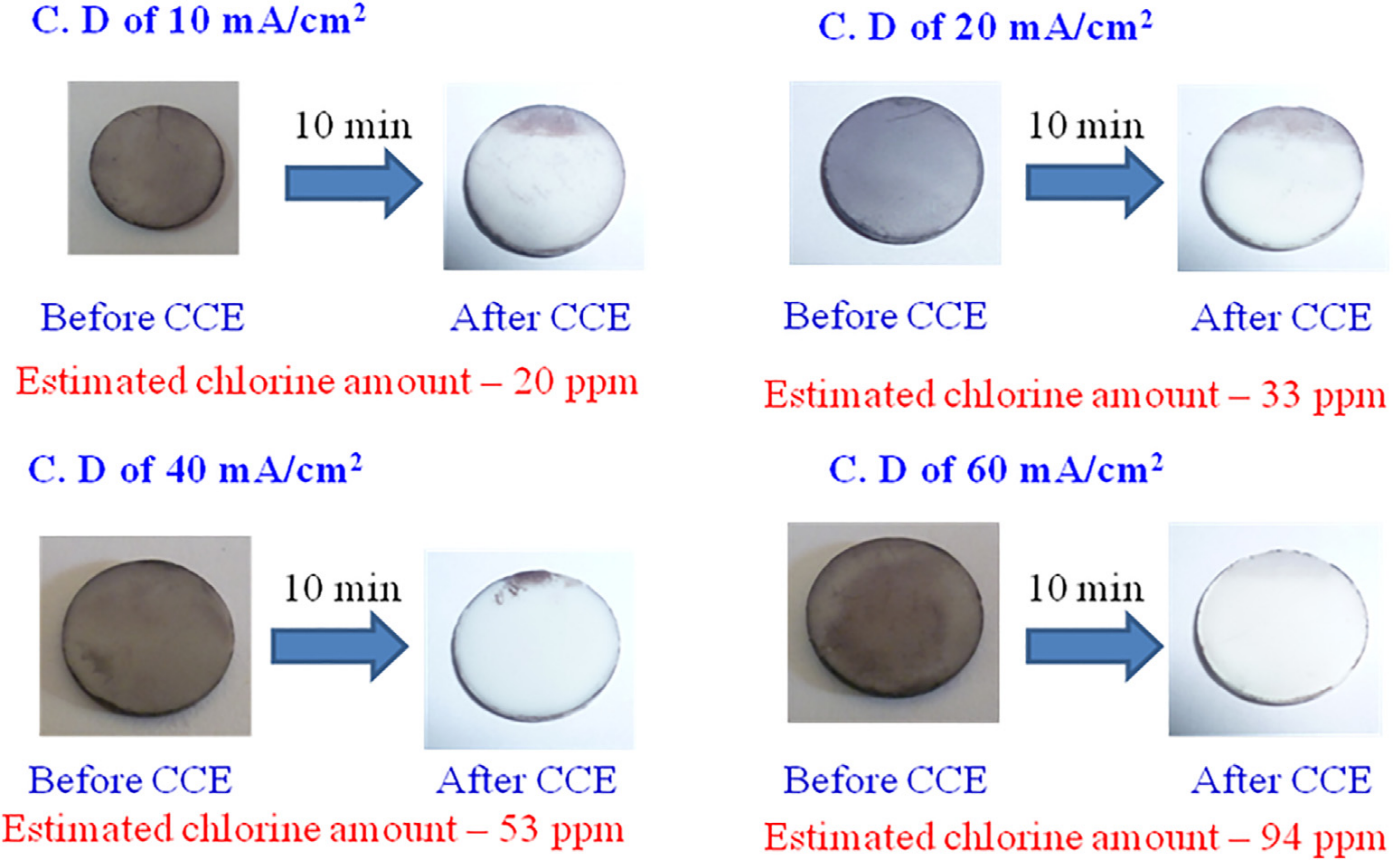
Photographic images obtained before and after CCE of Fe-TA-HAp at different current densities in phosphate buffer containing 0.1 M NaCl for 10 min. Surface area of Pt is 6.0 cm^2^.

When the surface area of the Pt electrode was increased from 1 to 6 cm^2^, the time required to remove the stain decreased to 15 min using an applied current density of 60 mA/cm^2^; and the estimated concentration of chlorine generated was lower, 88.6 ppm (Figure 5). For the optimization studies, CCE experiments were next carried out at different current densities ranging from 10-60 mA/cm^2^ with 0.1 M NaCl for 10 min on Pt (6 cm^2^) (Figure 6). At the lowest current density (10 mA/cm^2^), the amount of chlorine generated was 20 ppm which increased with increasing current densities. From the above experiments, the optimum current density was taken as 10 mA/cm^2^.

**Figure 7:**
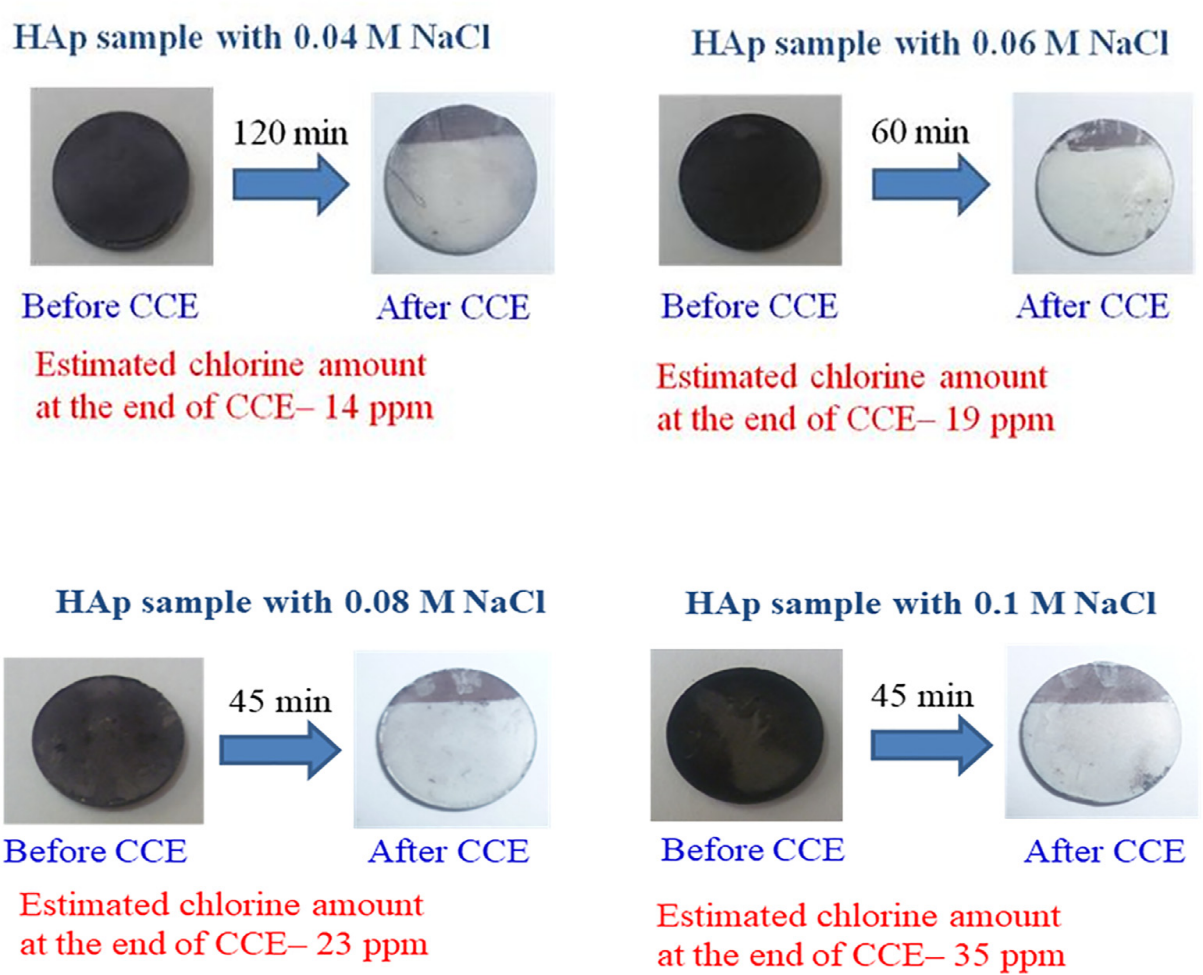
Photographic images obtained before and after CCE of Fe-TA-HAp in phosphate buffer containing different concentrations of NaCl. Surface area of Pt is 6.0 cm^2^ and current density is 10 mA/cm^2^.

Electrolysis experiments were carried out to optimize the concentration of NaCl for obtaining the minimum chlorine content needed to clearly see an improvement in stain removal over a 15–30 min period. Figure 7 shows photographic images obtained after CCE of Fe-TA-stained HAp carried out at different times using different concentrations of NaCl at an optimized current density of 10 mA/cm^2^. With increased NaCl concentrations, the time for stain removal decreased, and the chlorine concentration similarly increased. For consumer applicability, lower chlorine concentrations were desirable to minimize any negative taste impact.

CCE was also carried out in 0.04 M and 0.1 M NaCl to measure the stain removal when applied to Fe-TA-BE. Over a period of nearly 50 min, 60 ppm of chlorine was generated in 0.1 M NaCl (compared to 35 ppm for Fe-TA-HAp) which was reduced to 40 ppm in 0.04 M of NaCl over the course of 100 min (compared to 14 ppm of chlorine generated within a 2 h time period for Fe-TA-HAp). Thus, more chlorine was generated for iron tannate stained BE vs. HAp samples. This observation may be associated with the increased roughness and hence higher stained surface area of the BE samples, which would lead to the consumption of more chlorine.

### 3.3 CCE in aqueous NaCl through the non-continuous process

**Figure 8:**
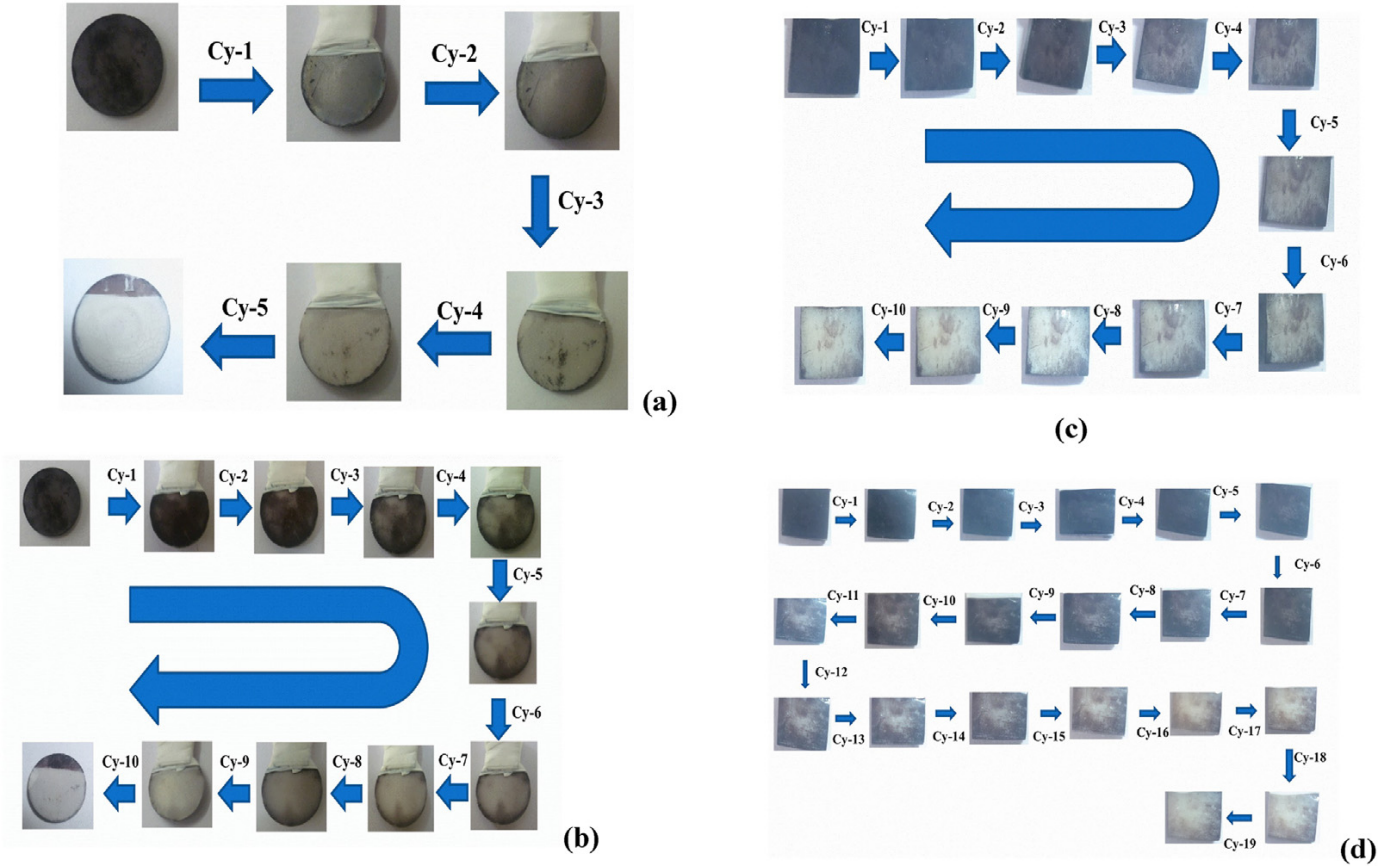
Photographic images obtained before and after CCE of different tooth models in phosphate buffer containing different concentrations of NaCl, Current density = 10 mA/cm^2^.Surface area of Pt is 6.0 cm^2^ a) Fe-TA-HAp in 0.1 M NaCl b) Fe-TA-HAp in 0.06 M NaCl, c) Fe-TA-BE in 0.1 M NaCl and d) Fe-TA-HAp in 0.06 M NaCl.

In order to further lower the amount of chlorine formed during the CCE experiments while maintaining maximum destaining efficacy, the electrolysis was conducted non-continuously in two different concentrations of NaCl namely 0.06 and 0.1 M, where the electrolyte solution was changed every 2, 3, 5 or 10 min. The amount of chlorine was too low to efficiently remove the stains when the solution was changed every 2, 3 and 5 min. More encouraging results were obtained when the electrolyte solution was changed every 10 min (Figure 8 and Table 3). The electrolysis was carried out on Pt electrodes having areas of 6 and 16 cm^2^ (cylindrical shape) for both Fe-TA-HAp and Fe-TA-BE samples.

**Table 3:** Amount of chlorine generated after CCE of Fe-TA-HAp carried out in 0.1 M NaCl in discontinuous process.

In 0.1 M NaCl for every 10 min interruption (Fe-TA-HAp)		In 0.1 M NaCl for every 10 min interruption (Fe-TA-BE)	
Cycle No	Conc. Cl_2_ (ppm)	Cycle No	Conc. Cl_2_ (ppm)
1	58	1	49
2	57	2	46
3	54	3	42
4	58	4	37
5	60	5	35
		6 7 8 9 10	39 39 40 55 58

For the non-continuous CCE studies of Fe-TA-HAp (Figs. 8a and b) and Fe-TA-BE samples (Figs. 8c and d) (6 cm^2^ Pt electrode area, 10 mA/cm^2^ current density), more chlorine was generated in less cycles when the experiment was conducted in 0.1 M NaCl vs 0.06 M NaCl (Table 3). The amount of chlorine generated for Fe-TA-HAp was higher than that detected during the continuous CCE process, irrespective of the NaCl concentration (compare 30 min times in Table 2 and cumulation of 30 min in Table 3). More chlorine was also generated for Fe-TA-HAp samples vs Fe-TA-BE samples. This result was perhaps observed because the iron tannate stained BE samples had more scratches and a more roughened surface (Figs. 8c and d) than the smoother hydroxy apatite discs. It thus was more difficult for the resulting hypochlorite to oxidize the less accessible stain areas resulting in more chlorine being generated and measured.

It is important to note that the CCE results carried out in both open and closed systems in 0.06 M of NaCl, for 10 min at a current density of 10 mA/cm^2^, in the absence and presence of substrate sample show that about 54% of chlorine escapes from the reaction cell and 30% of chlorine only reacts with the sample. Further, it is understandable that the estimated chlorine content is very high for those partially stain removed samples. This is due to the fact that the amount of stain is only minimal and the produced chlorine does not have enough stain to react. As a result, the chlorine amount is increasing. The effect of distance between the working electrode and the sample has only less effect on stain removal.

The final experimental design involving CCE of Fe-TA-HAp and Fe-TA-BE samples was carried out using electrodes with high surface areas (i.e., cylindrical electrode, area of 16 cm^2^) at low current densities of 0.5 mA/cm^2^ in 0.06 M of NaCl. Under these experimental conditions, the lowest amount of chlorine was generated, 13 ppm after completing 34 cycles.

### 3.4 Ion chromatographic analysis

Ion chromatography analysis was done to measure the products generated during continuous and non-continuous CCE in the presence and absence of a stained substrate in NaCl solution. Based on the IC analysis results, a very low amount of sodium perchlorate was detected during the non-continuous process irrespective of the presence of stained substrate. Significantly more chlorates were generated for Fe-TA-HAp (140 ppm) and Fe-TA-BE (37 ppm) during the continuous process. Sodium chlorite was not detected in any CCE experiments.

### 3.5 Constant potential electrolysis (CPE) experiments without samples

Experiments based on CPE were also carried out in the absence of stained substrates in order to determine the maximum amount of chlorine that could be generated at the end of the reaction. CPE was carried out in NaCl (0.06 M and 0.1 M) phosphate buffered solution (pH of 5, 7, 9) at fixed voltages of 1.5 V, 2.0 V and 3.0 V for one and three minutes on three electrode systems containing Pt as working (1 cm^2^), counter and reference. Table 4 shows the amount of chlorine and the current generated with the specified potential. The results show that at NaCl concentrations of 0.06 M or 0.1 M, the output current as well as the amount of chlorine generated decreased from pH 5 to pH 7 and then increased from pH 7 to pH 9. At all pH values evaluated, the amount of chlorine increased with an increase in applied potential as a result of an increase in current. The present study clearly reveals that the electrochemical method involving non-continuous CCE experiments carried out in NaCl solution on Pt electrode can remove iron tannate stains effectively both on BE and HAP, which is an alternate approach to applying a peroxide treatment. The time for stain removal and hypochlorite content can be further minimized by employing large areas and using at low current densities.

**Table 4:** Amount of chlorine generated after CPE of Fe-TA-HAP carried out at different pH for one minute in NaCl solution.

	pH = 5				pH = 7				pH = 9			
	Conc. of NaCl											
	0.06M		0.1 M		0.06M		0.1 M		0.06M		0.1 M	
Voltage (V)	1.5	2.0	1.5	2.0	1.5	2.0	1.5	2.0	1.5	2.0	1.5	2.0
Current (mA)	37	131	36	77	12	47	15	43	27	159	25	157
Cl (ppm)	42	60	32	40	23	32	22	30	49	68	35	42

## 4 Discussion

**Figure 9:**
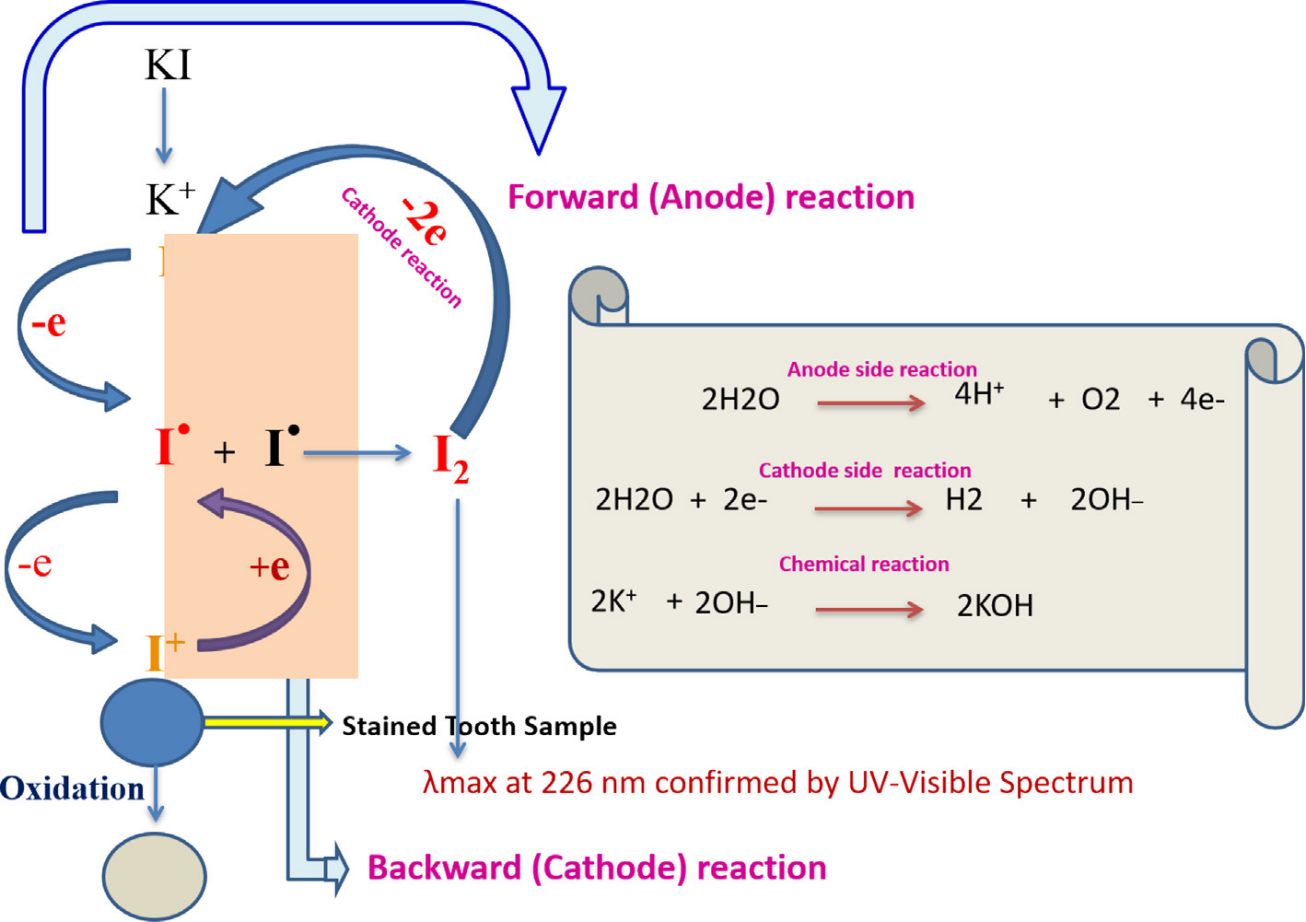
Reaction mechanism involving the CCE in KI.

The results presented in this manuscript demonstrate that tooth stains can be removed through CCE processes. Simple electrolyte solutions (e.g. KI, KCl, and NaCl) were used to generate active free radicals that oxidize stains on representative tooth models: iron tannate stained HAp and bovine enamel. After completing numerous CCE experiments, the optimal Pt electrode size, current density, electrolyte solution concentration, and experimental design were identified. The results suggest that the use of NaCl and KCl solutions are preferred over KI solutions since no new stain is produced during the electrolysis process. Iodine was measured by iodometric titration at the end of CCE experiments in KI and was found to be in the range of 30 ppm for Fe-TA-HAp samples treated with 0.1 M of KI and nearly 40 ppm in 0.2 M KI. UV-visible spectral studies confirm the formation of I_2_ in the reaction mixture to be the source of the yellow color observed at the end of the CCE experiments (Figure 3). The electrochemical reaction mechanism involves the one electron reduction of iodine anion to iodine free radical followed by another one electron reduction generating iodonium cation (Figure 9). Iodine free radicals also combine to form an iodine molecule. This iodonium cation oxidizes the Fe-tannate stain on tooth samples, reducing the appearance of the iron tannate stain. The reversible reaction also leads to the formation of iodine free radical and iodine ion. Other side reactions include oxygen evolution and the formation of hydroxyl ions resulting in the observed increase in pH. Since, the time duration of destaining in KI medium is high, further experiments were oriented towards conducting CCE in KCl and NaCl solutions.

Although less chlorine was generated in NaCl vs KCl solution (under equivalent CCE conditions), the rate of bleaching was greater for NaCl. CCE of Fe-TA-stained HAp in NaCl solution reveals that at the lowest current density of 10 mA/cm^2^, the amount of hypochlorite generated was 20 ppm on the Pt electrode with area of 6 cm^2^. For the purposes of the described research, the objective was to generate as little hypochlorite as possible while retaining an effective stain removing capability. CCE experiments were carried out for Fe-TA-HAp and Fe-TA-BE on a 16 cm^2^ cylindrical Pt electrode at a current density of 0.5 mA/cm^2^ and 1.0 mA/cm^2^ respectively, in 0.06 M NaCl. A non-continuous process was followed changing the solution every 10 min. Using this design, the lowest hypochlorite concentration of 13 ppm was measured after 34 cycles. IC analysis was used to identify other chlorine side products. None were detected for the non-continuous process. However, for the continuous process, a very low amount of sodium perchlorate was generated, (37 ppm for Fe-TA-BE and 140 ppm for Fe-TA-HAp for the CCE. Sodium chlorite was not detected in any of the CCE experiments. CPE in absence of samples revealed that at both concentrations of NaCl (0.06 M or 0.1 M) evaluated, the output current as well as the amount of chlorine generated decreased from pH 5 to pH 7 and then increased from pH 7 to pH 9. At each pH (5 or 7 or 9), the amount of chlorine increased with an increase in the applied potential as a result of an increase in current.

**Figure 10:**
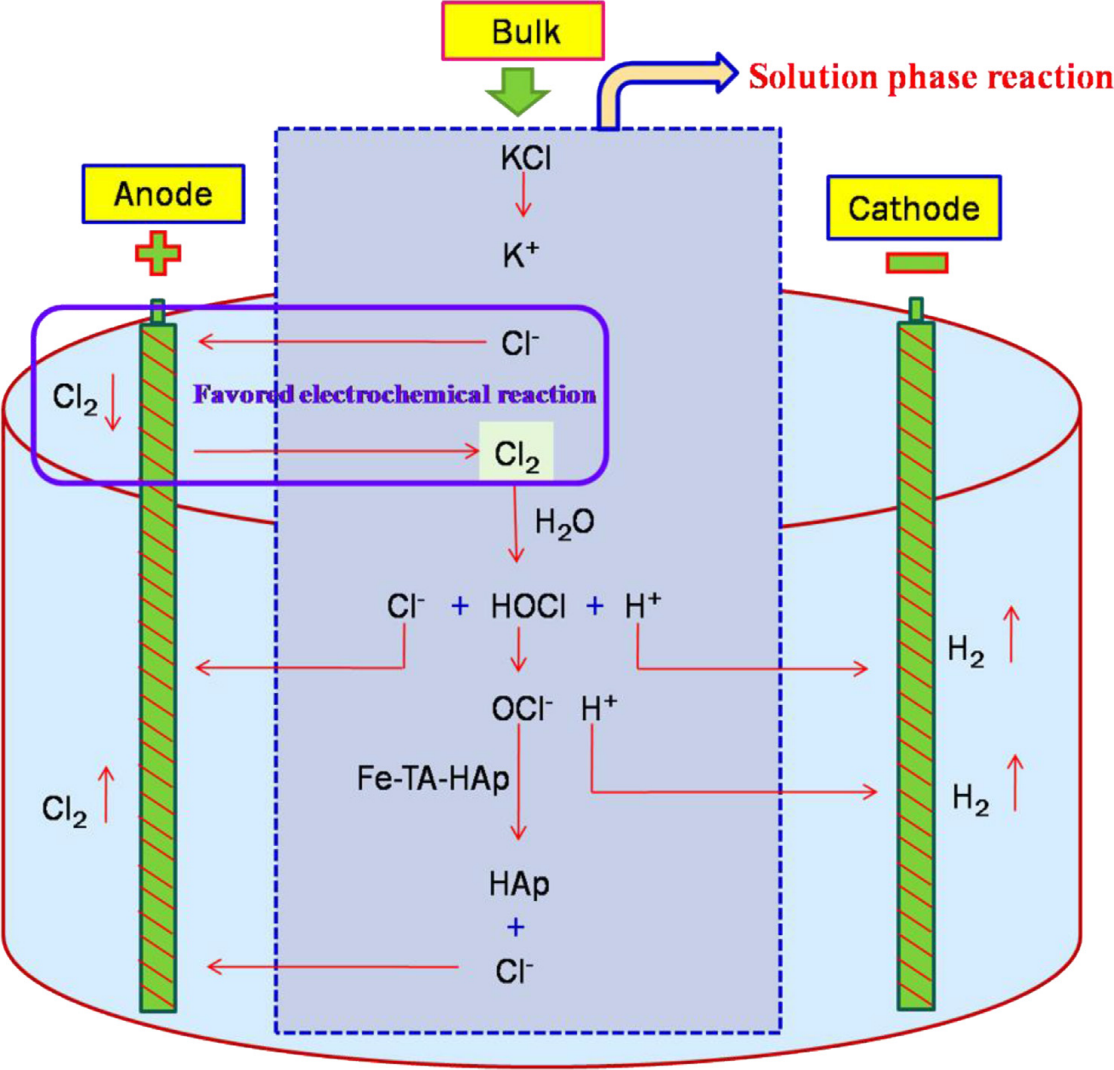
Reaction mechanism involving the CCE in KCl/NaCl.

The mechanism driving the stain removal of Fe-TA-stained HAp in chloride medium (Figure 10) is totally different from the electro oxidation mechanism involving KI (Figure 9). For chlorine salts, chlorine ions are oxidized at the anode to form chlorine gas which dissolves in water to form hypochlorous acid. The hypochlorous acid dissociates to hypochlorite ion and the proton generated is reduced at the cathode to form hydrogen gas. The hypochlorite ion oxidizes the Fe-TA on HAp and BE, eliminating the stain. This chloride bleaching mechanism has previously been discussed in earlier publications [29, 30]. The oxidative chemistry of iron tannate not being very simple, involves the formation of complex products. Since this study is a preliminary one, the research work is restricted to stain removal only at a lowest concentration of hypochlorite. The in-depth discussions on the methodology and characterization of various products at different current densities, concentration of substance under galvanostatic vs potentiostatic electrolysis is under progress.

This present work demonstrates the effective bleaching of different tooth models stained with Fe-TA by galvanostatic electrolysis in brine solution. As a next step, these electrochemical stain removal processes should be extended to naturally stained human teeth samples. Human teeth are usually stained not only with tannin, but with different chromophores such as caffeine, resveratrol, flavones, flavanone etc., Hence, different experimental parameters such as current density, concentration of electrolyte, area of working electrode etc., would need to be optimized in order to attain effective oxidation of these other chromophores. Further, the micro reactor cell set-up should be fabricated with the involvement of microelectrodes so that the stain removal may be carried out directly on the human teeth by applying minimum current. This will pave the way for the lowest quantity of supporting electrolytes needed for electrolysis resulting in a safe and effective amount of hypochlorite generation during the course of the reaction.

## 5 Conclusions

In this work, CCE (using KI, KCl, and NaCl solutions) was evaluated to determine the effectiveness of removing Fe-TA stains from bovine enamel and HAp tooth models. Based on the results, the chloride salts performed better than KI. Higher concentrations, larger surface area electrodes, and higher current densities resulted in greater stain removal. For KI, another chromophore was generated that was also colored and for the chloride salts, unsafe levels of chloride by products were generated. In order to maximize the effectiveness of stain removal while staying within the safety limits, the CCE experiment was conducted by replacing the halide solution every 10 min (e.g., discontinuous process). The final optimal experimental design used a 16 cm^2^ Pt electrode at a current density of 0.5 mA/cm^2^ with the concentration 0.06 M of NaCl. After 34 cycles (of replacing electrolyte solution ever 10 min), 13 ppm of hypochlorite was produced and the Fe-TA stain was effectively removed from both bovine enamel and HAp samples. In comparison, under similar conditions but with a continuous process (ie no solution replacement), considerable quantities of chlorate appeared for Fe-TA-BE (37 ppm) and Fe-TA-HAp (140 ppm) samples. Electrooxidation of Fe-TA by KCl and KI occurs through the generation of hypochlorite and iodonium ion (I^+^) respectively.

## Declarations

### Author contribution statement

Vembu Suryanarayanan: Conceived and designed the experiments; Wrote the paper.

Deepak Kumar Pattanayak, LaTonya Kilpatrick: Conceived and designed the experiments.

Rethinam Senthil Kumar, Lin Fei: Performed the experiments.

Suman Chopra, GuoFeng Xu: Analyzed and interpreted the data.

Cajetan Dogo-Isonagie, Patrik Johansson: Contributed reagents, materials, analysis tools or data.

### Funding statement

Dr. Pattanayak Deepak Kumar was supported by 10.13039/100004368Colgate-Palmolive Company (SSP 21/16).

### Data availability statement

The data that has been used is confidential.

### Declaration of interests statement

The authors declare no conflict of interest.

### Additional information

No additional information is available for this paper.
